# Unsupervised Machine Learning of MRI Radiomics Features Identifies Two Distinct Subgroups with Different Liver Function Reserve and Risks of Post-Hepatectomy Liver Failure in Patients with Hepatocellular Carcinoma

**DOI:** 10.3390/cancers15123197

**Published:** 2023-06-15

**Authors:** Qiang Wang, Changfeng Li, Geng Chen, Kai Feng, Zhiyu Chen, Feng Xia, Ping Cai, Leida Zhang, Ernesto Sparrelid, Torkel B. Brismar, Kuansheng Ma

**Affiliations:** 1 Division of Medical Imaging and Technology, Department of Clinical Science, Intervention and Technology (CLINTEC), Karolinska Institutet, 141 86 Stockholm, Sweden; qiang.wang@ki.se (Q.W.); torkel.brismar@ki.se (T.B.B.); 2 Division of Radiology, Department of Clinical Science, Intervention and Technology (CLINTEC), Karolinska Institutet, Karolinska University Hospital, 141 86 Stockholm, Sweden; 3 Institute of Hepatobiliary Surgery, Southwest Hospital, Army Medical University, Chongqing 400038, China; changfeng.li2020@outlook.com (C.L.); fengkai@tmmu.edu.cn (K.F.); chenzhiyu_umn@163.com (Z.C.); txzzxf@163.com (F.X.); zhangleida@tmmu.edu.cn (L.Z.); 4 Department of Hepatobiliary Surgery, Daping Hospital, Army Medical University, Chongqing 400042, China; 5 Department of Radiology, Southwest Hospital, Army Medical University, Chongqing 400038, China; pingc_ddd@sina.com; 6 Division of Surgery, Department of Clinical Science, Intervention and Technology (CLINTEC), Karolinska Institutet, Karolinska University Hospital, 141 86 Stockholm, Sweden; ernesto.sparrelid@ki.se

**Keywords:** radiomics, magnetic resonance imaging, machine learning, liver function, hepatocellular carcinoma

## Abstract

**Simple Summary:**

The liver function reserve of patients with hepatocellular carcinoma (HCC) is heterogeneous. The preoperative accurate evaluation of liver function has a vital role in the prevention of unfavorable postoperative complications such as post-hepatectomy liver failure. In this study, unsupervised clustering analysis of radiomics features extracted from preoperative gadoxetic-acid-enhanced MRIs was performed for liver function stratification on 276 HCC patients. Two distinct subgroups were identified (i.e., subgroups 1 and 2). Subgroup 2 had impaired liver function as presented by older age, more albumin–bilirubin grades 2 and 3, and a higher indocyanine green retention rate than that of subgroup 1 (all *p* < 0.05). Compared with subgroup 1, subgroup 2 was associated with a higher risk of postoperative liver failure, postoperative complications, and longer hospital stays (all *p* < 0.05). Our findings indicate the potential for the use of radiomics features based on preoperative gadoxetic-acid-enhanced MRI for noninvasive liver function assessment in HCC patients.

**Abstract:**

Objective: To identify subgroups of patients with hepatocellular carcinoma (HCC) with different liver function reserves using an unsupervised machine-learning approach on the radiomics features from preoperative gadoxetic-acid-enhanced MRIs and to evaluate their association with the risk of post-hepatectomy liver failure (PHLF). Methods: Clinical data from 276 consecutive HCC patients who underwent liver resections between January 2017 and March 2019 were retrospectively collected. Radiomics features were extracted from the non-tumorous liver tissue at the gadoxetic-acid-enhanced hepatobiliary phase MRI. The reproducible and non-redundant features were selected for consensus clustering analysis to detect distinct subgroups. After that, clinical variables were compared between the identified subgroups to evaluate the clustering efficacy. The liver function reserve of the subgroups was compared and the correlations between the subgroups and PHLF, postoperative complications, and length of hospital stay were evaluated. Results: A total of 107 radiomics features were extracted and 37 were selected for unsupervised clustering analysis, which identified two distinct subgroups (138 patients in each subgroup). Compared with subgroup 1, subgroup 2 had significantly more patients with older age, albumin–bilirubin grades 2 and 3, a higher indocyanine green retention rate, and a lower indocyanine green plasma disappearance rate (all *p* < 0.05). Subgroup 2 was also associated with a higher risk of PHLF, postoperative complications, and longer hospital stays (>18 days) than that of subgroup 1, with an odds ratio of 2.83 (95% CI: 1.58–5.23), 2.41(95% CI: 1.15–5.35), and 2.14 (95% CI: 1.32–3.47), respectively. The odds ratio of our method was similar to the albumin–bilirubin grade for postoperative complications and length of hospital stay (2.41 vs. 2.29 and 2.14 vs. 2.16, respectively), but was inferior for PHLF (2.83 vs. 4.55). Conclusions: Based on the radiomics features of gadoxetic-acid-enhanced MRI, unsupervised clustering analysis identified two distinct subgroups with different liver function reserves and risks of PHLF in HCC patients. Future studies are required to validate our findings.

## 1. Introduction 

Hepatocellular carcinoma (HCC) ranks as the fourth most common cause of cancer-related mortality globally, with a median survival ranging from 6 to 10 months [1,2]. A host of therapies are available for HCC treatment, including traditional surgical resection, ablation, transplantation, interventional therapy, and newly emerged molecular targeted therapy and immunotherapy [3]. Among these options, liver resection remains a cornerstone in the treatment of HCC. Before liver resection, a comprehensive and accurate evaluation of the liver function reserve is essential to ensure a safe surgery and to avoid unfavorable postoperative complications, such as post-hepatectomy liver failure (PHLF) [4]. PHLF, which is the leading cause of perioperative mortality and hence is a feared complication, has an approximate incidence of 10–30% [5,6]. Except for supportive care or liver transplantation, there are no effective therapies for the treatment of PHLF [4]. In order to avoid surgery in patients with too high risk of PHLF, it is crucial to thoroughly assess the liver function reserve before liver resection. This issue is unfortunately often a clinical reality, given that HCC is often developed at the basis of underlying liver disease (such as hepatitis B/C virus infection or alcohol abuse) and liver fibrosis/cirrhosis [7]. In these patients, the liver function reserve is chronically undermined. In addition, the distribution of liver function among the different liver segments might be uneven [8]. 

Traditional approaches used for preoperative assessment of liver function reserve include serum biochemical tests (such as aspartate/alanine transaminase, albumin, bilirubin, and prothrombin time) and clinical scoring systems (for instance, the Child–Pugh scoring system and the model for end-stage liver disease (MELD) system). In surgical oncology, the indocyanine green (ICG) test is also a widely applied approach for the quantitative evaluation of liver function reserve [9]. ICG is an inert dye that is almost exclusively extracted by the hepatocytes after intravenous injection and excreted into the bile without biotransformation [10]. It is a well-established test and has been incorporated in several guidelines used for the recommendation of treatment options and liver resection extent, especially in Asian countries [11]. The two common parameters in the ICG test are the retention rate at 15 min after administration (ICG-R15, %) and the plasma disappearance rate (ICG-PDR, %/min) [10]. 

Gadoxetic acid is a liver-specific contrast medium for magnetic resonance imaging (MRI). It is commonly used for the detection, diagnosis, and characterization of hepatic lesions in clinical practice [12]. In recent years, an extensive body of studies has demonstrated that gadoxetic-acid-enhanced MRI can be applied to evaluate liver function and to estimate the risk of PHLF by quantifying the signal intensity of the hepatic parenchyma or by measuring the T1 relaxometry [13,14,15,16]. The images used are from the hepatobiliary phase, i.e., 10–40 min after gadoxetic acid administration, in which the uptake and excretion of the gadoxetic acid by the hepatocytes reaches an equilibrium [17]. Parameters derived from gadoxetic-acid-enhanced hepatobiliary phase MRIs have shown a close correlation with the Child–Pugh score, MELD score, and ICG tests [15,18]. When predicting the risk of PHLF, the efficacy of gadoxetic-acid-enhanced MRI even outperforms the ICG tests [14]. 

In the past decades, various high-throughput techniques have been employed for the investigation of liver cancers, including transcriptomics, proteomics, epigenetics, and phenomics [19,20,21]. This field has also witnessed the emergence of a novel technique called radiomics, which extracts high-throughput imaging features from daily used images, such as MRI [22]. While traditional “omics techniques” have provided valuable insights into the molecular profiles and biological processes involved in tumorigenesis, they often require invasive sample collection methods or extensive laboratory procedures. In contrast, radiomics harnesses the power of commonly used imaging modalities to extract quantitative features [22,23]. These radiomics features encompass a wide range of information, including tumor shape, texture, intensity, and spatial relationships, offering a noninvasive and complementary approach to understanding liver tumor heterogeneity and predicting patient outcomes [22,23]. Radiomics holds great promise in the comprehensive analysis and characterization of liver cancers [24]. 

To date, a host of powerful and robust radiomics models have been developed by using various machine/deep-learning approaches, such as random forest, support vector machine, convolutional neural networks, transfer learning, and deep-learning architectures [25,26,27,28]. Yet, most techniques used in those studies can be categorized into supervised machine-learning techniques, in which the clinical outcomes have been artificially labeled. By contrast, unsupervised machine learning is a type of algorithm that detects cluster numbers, membership, and boundaries in an unlabeled dataset. Compared with supervised machine learning, unsupervised machine learning allows researchers to gain insights into the underlying data distribution, capture complex tumor/disease heterogeneity, identify novel biomarkers, and understand the inherent structures and relationships within the medical data [29].

The heterogeneity of the liver function reserve in HCC patients might also be reflected by the radiomics features of the gadoxetic-acid-enhanced MRI, but research using these features for liver function evaluation remains rare. Only a few studies using supervised machine learning have developed radiomics models for estimating ICG levels or predicting the risk of PHLF [30,31,32,33]. To date, there has not been any research exploring the role of unsupervised machine learning in the stratification of the liver function reserve. This study was therefore designed to use unsupervised machine learning to identify distinct subgroups of different liver function reserve in the radiomics features of the preoperative gadoxetic-acid-enhanced MRI in HCC patients who were scheduled for liver resection. The differences in the risk of PHLF, postoperative complications, and length of hospital stay were then evaluated between the subgroups.

## 2. Materials and Methods

### 2.1. Study Design and Patient Inclusion

This study was a secondary analysis of existing data that previously had been used to develop and internally validate a clinical-radiomics model for PHLF prediction. The research protocol was approved by the Institutional Review Board of the hospital, Army Medical University [No. (B)KY2021068]. The study was conducted in accordance with the Helsinki Declaration and the data were analyzed anonymously. Informed consent was waived due to the retrospective nature of this study. 

**Figure 1:**
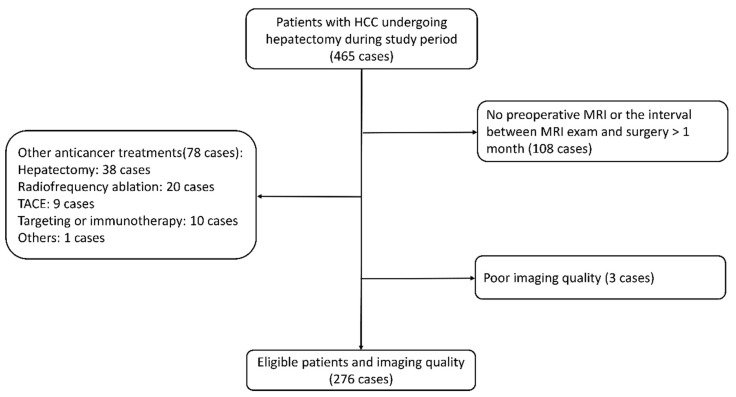
CONSORT flow diagram of this study. Note: HCC, hepatocellular carcinoma; MRI, magnetic resonance imaging; TACE, transarterial chemoembolization.

Consecutive patients who underwent liver resection between January 2017 and March 2019 and were diagnosed with HCC by the postoperative pathology exam were initially included. Exclusion criteria were: (1) Gadoxetic-acid-enhanced MRI was performed more than one month before liver resection; (2) Anti-cancer therapies were performed before liver resection; for instance, radiofrequency ablation, transarterial chemoembolization, hepatectomy, portal vein embolization, and systematic therapy; (3) Insufficient imaging quality, such as motion artifacts and obvious noise. A total of 276 eligible patients were eventually included in this study. The study CONSORT flow diagram is provided in Figure 1.

### 2.2. Clinical Variables

The following clinical variables were collected: demographic and baseline information (including age, gender, and body mass index); chronic liver disease (etiology and cirrhosis); tumor size (≤5 or >5 cm); surgery and intraoperative data, including resection extent (minor if resected segments < 3 or major if segments ≥ 3) [34], laparoscopic surgery (yes or no), operative time (≤60 min or >60 min), and estimated blood loss (≤400 or >400 mL); laboratory tests, including platelet count (≤125 or >125 × 10^9^/L), aspartate transaminase (AST) (≤42 or >42 IU/L), and alanine transaminase (ALT) (≤42 or >42 IU/L); Child–Pugh grade; MELD score (≤9 or >9); albumin–bilirubin (ALBI) score (the results were categorized into Grades 1, 2, and 3; Grade 2 and Grade 3 were merged into “Grade 2/3” in this study); ICG tests (including ICG-R15 and ICG-PDR); and length of hospital stay (dichotomized by the median, i.e., ≤18 or >18 days). 

PHLF was diagnosed in accordance with the International Study Group of Liver Surgery definition: an increased international normalized ratio and hyperbilirubinemia (above the local laboratory’s normal range) on postoperative day 5 or later [35]. Postoperative complications were graded by applying the Clavien–Dindo classification, with grade ≥ II as significant complications. 

### 2.3. Gadoxetic-Acid-Enhanced MRI Exam

All patients underwent their MRI exam on a 3.0 T scanner (Magnetom Trio, Siemens Healthcare, Germany). A T1-weighted 3D volume interpolated breath-hold exam sequence was used to acquire dynamic contrast-enhanced images before, at the time of aorta enhancement, ~60 s, and 15 min after contrast media administration, corresponding to the unenhanced, arterial, portal venous, and hepatobiliary phase, respectively [36]. Gadoxetic acid (0.1 mg/kg body weight, Primovist^®^, Bayer Pharma, Berlin, Germany) was administered through an antecubital vein followed immediately by a 20 mL saline flush. The detailed scanning parameters at each phase are provided in Supplementary Table S1. 

**Figure 2:**
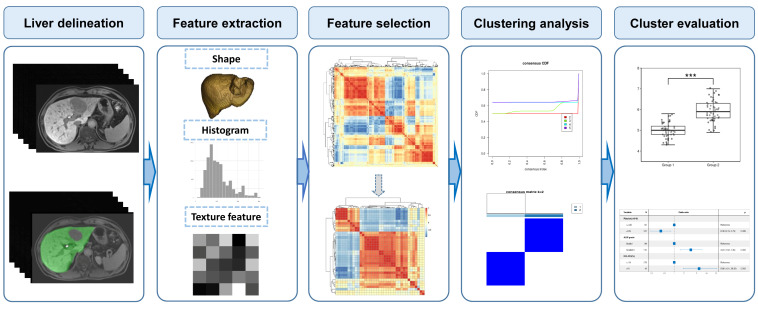
The workflow of this study using unsupervised clustering analysis on radiomics features extracted from the preoperative gadoxetic-acid-enhanced MRI for stratification of liver function reserve in patients with hepatocellular carcinoma.

After acquisition of the MR images, the workflow of this research consisted of five steps: liver delineation, feature extraction, feature selection, clustering analysis, and cluster comparison (Figure 2). 

### 2.4. Liver Delineation and Radiomics Feature Extraction 

**Figure 3:**
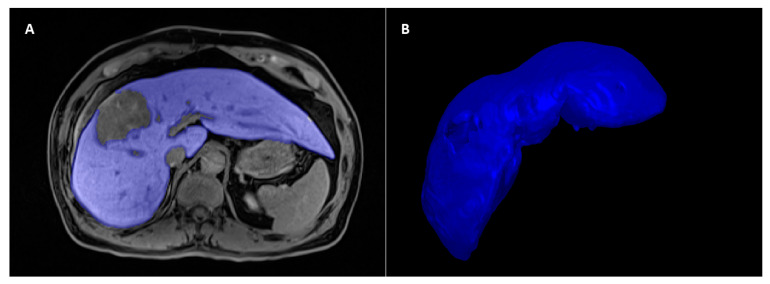
The volume of interest of the non-tumoral hepatic parenchyma was delineated at the hepatobiliary phase image of the preoperative gadoxetic-acid-enhanced MRI (the blue part). (**A**) Cross-sectional imaging; (**B**) 3D visualization of the segmented volume of interest.

The volume of interest (VOI) of the non-tumoral hepatic parenchyma at the hepatobiliary phase images was manually delineated by one researcher (C.L., with 3 years’ experience of abdominal imaging) using the software ITK-SNAP (http://www.itksnap.org/, accessed on 1 July 2022) (Figure 3). Before feature extraction, the images were resampled into a voxel size of 1 × 1 × 1 mm^3^, and the intensity histogram-bin width was fixed at 25. Radiomics features were extracted from the VOI by using the Python package “PyRadiomics” (https://github.com/AIM-Harvard/pyradiomics, accessed on 20 August 2022), including the following categories of features: (1) Shape (2D and 3D) (*n* = 14), (2) First-order statistics (*n* = 18), (3) Gray level co-occurrence matrix-derived feature (*n* = 24), (4) Gray level run length matrix-derived feature (*n* = 16), (5) Gray level size zone-derived feature (*n* = 16), (6) Gray level dependence matrix-derived feature (*n* = 14), (7) Neighboring gray tone difference matrix feature (*n* = 5). In total, 107 radiomics features were extracted. Radiomics features extracted by “PyRadiomics” are consistent with the Image Biomarker Standardization Initiative [37].

### 2.5. Radiomics Feature Selection

To evaluate the interobserver agreement, interclass correlation coefficient (ICC) analysis was performed on 30 randomly selected cases that were delineated by two researchers (C.L and P.C., with 3 and 20 years of abdominal MRI diagnosis experience, respectively). Radiomics features with an ICC greater than 0.75 were considered reproducible [38]. Spearman correlation analysis of the reproducible radiomics features was then performed to reduce redundancy, with one feature in all pairs with a correlation coefficient greater than 0.99 randomly abandoned. 

### 2.6. Unsupervised Clustering Analysis

An unsupervised machine learning algorithm, consensus clustering analysis [39], was applied to identify the clinical subtypes of patients based on the filtered radiomics features. Consensus clustering analysis applies a subsampling technique to induce variability, and then it calculates the stability of the clusters (“consensus”) under multiple iterations of a specific clustering algorithm on the subsamples [40]. It can distinguish samples into several subtypes by using a predefined number of clusters (k), so as to discover new disease subtypes or perform a comparative analysis of different clusters [39]. It is a robust and commonly used approach in cancer genetic research [41]. 

After the feature data were normalized with *z*-score normalization, the R package “ConsensusClusterPlus” (https://bioconductor.org/packages/ConsensusClusterPlus/, accessed on 1 September 2022) was used to perform the consensus clustering analysis [42], with the key parameters settings as follows: clusters (k) range: 2 to 5; proportion of items to subsampling: 80%; number of subsampling: 200; cluster algorithm: hc (hierarchical clustering); and distance: Canberra; all other parameters were set to default. The optimal clustering number (k) was determined by comprehensively evaluating the consensus matrix heat map, cumulative distribution function, and cluster-consensus scores.

### 2.7. Cluster Comparison and Statistical Analysis 

Continuous variables were expressed as median with range and compared using the Mann–Whitney *U* test. Categorical variables were presented as numbers and percentages, and their differences were detected with the chi-squared test or Fisher’s exact test. The odds ratio (OR) of the different liver function subgroups, categorized by their Child–Pugh grade, MELD score, ALBI grade, and our unsupervised clustering analysis, for the PHLF risk, significant postoperative complications, and length of hospital stay, was calculated and presented in a forest plot for intuitive comparison. A two-tailed *p*-value < 0.05 was regarded as statistically significant and a *p*-value < 0.10 as a tendency. All statistical analyses were conducted on R software (version 4.0.2, R Foundation for Statistical Computing, Vienna, Austria).

## 3. Results

### 3.1. Basic Characteristics of the Entire Cohort

The overall cohort (*n* = 276) had a predominance of males (86.2%) and patients aged ≤55 years (71.4%). Most patients had hepatitis B virus infection (76.8%), and around half the patients had cirrhosis (52.9%). The proportion of tumor size ≤5 and >5 cm was roughly equal (47.1% vs. 52.9%). A majority of patients received a minor liver resection (71%). A majority of patients had a Child–Pugh Grade A (98.6%) or a MELD score ≤ 9 (93.8%), while the number of patients with an ALBI Grade 1 was roughly equal to those with Grade 2/3 (45.7% vs. 54.3%). The median ICG-R15 test was 3.7%, with a range from 0.3% to 33.5%, and the median ICG-PDR was 21.9%/min (range 7.3%/min–39.4%/min). The incidence of PHLF was 23.6%, and 12.7% of patients developed significant postoperative complications (Clavien–Dindo classification ≥ grade II). 

### 3.2. Radiomics Feature Selection and Unsupervised Clustering Analysis 

**Figure 4:**
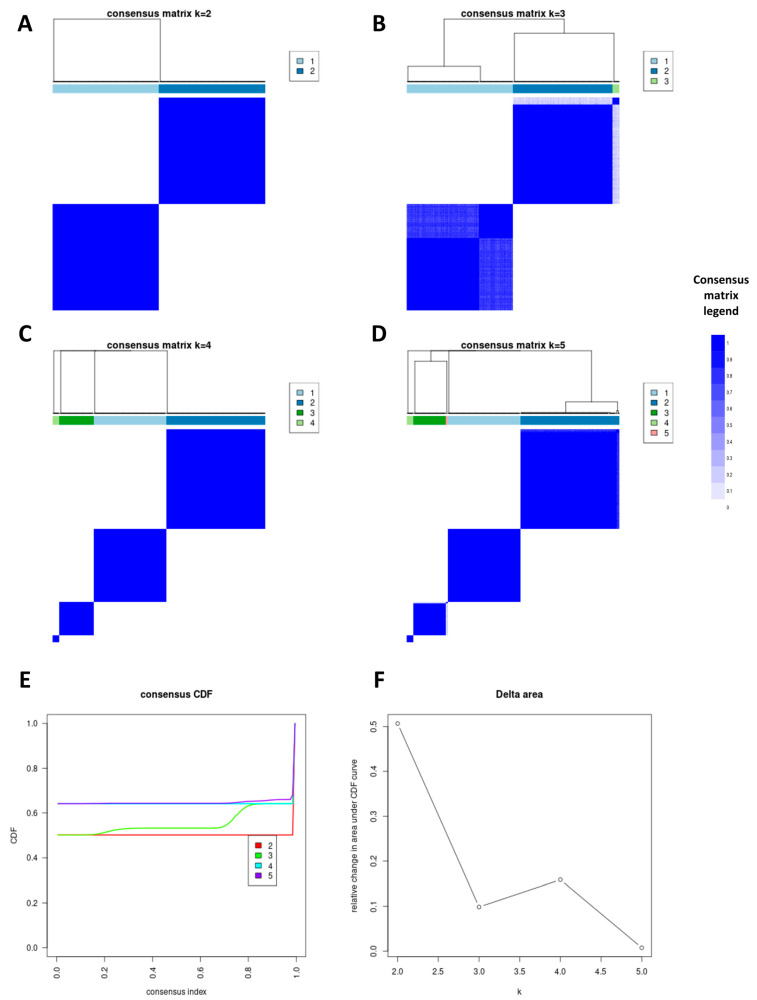
(**A**–**D**) Consensus matrix heat map shows the consensus values when the clustering number (k) was set as 2 (**A**), 3 (**B**), 4 (**C**), and 5 (**D**). (**E**) Cumulative distribution function plot depicts consensus distribution of each clustering number (k). (**F**) Delta area plot shows the relative changes of the area under the cumulative distribution function curve.

Among the 107 radiomics features extracted from the hepatobiliary phase, 37 reproducible and non-redundant features remained after the removal of features with ICC less than 0.75 and a correlation coefficient of more than 0.99. They consisted of 2 morphological features, 8 first-order statistical features, and 27 textual features (Supplementary Table S2). These features were then fed into the consensus clustering algorithm, and it assigned a grouping number to each patient. It finally yielded 2, 3, 4, and 5 subgroups according to the preset clustering number (k) (Figure 4). 

By examining the consensus matrix heat map, cumulative distribution function plot, and cluster-consensus values, the most stable results were observed when the clustering number (k) was set to 2. Coincidentally, the two subgroups had an equal number of patients (each *n* = 138). 

### 3.3. Subgroup 1 vs. Subgroup 2

Subgroup 1 had significantly more patients with younger age (≤55 years) (79.7% vs. 63.0%, *p* < 0.05) and a tendency to less cirrhosis (47.1% vs. 58.7%, *p* = 0.07) in comparison with subgroup 2. Better liver function reserve was observed in subgroup 1 than in subgroup 2, with significantly lower ALBI Grade 2/3 (46.4% vs. 62.3%, *p* < 0.05), and ICG-R15 (3.2% vs. 4.1%, *p* < 0.05), while ICG-PDR was significantly higher (22.9%/min vs. 21.1%/min, *p* < 0.05). Detailed information is provided in Table 1. 

Subgroup 2 had a higher incidence of PHLF and significant postoperative complications than subgroup 1 (32.6% vs. 14.5% and 17.4% vs. 8.0%, respectively, both *p* < 0.05). More patients in subgroup 2 had a long hospital stay (>18 days) than the patients in subgroup 1 (58.7% vs. 39.9%, *p* < 0.05).

### 3.4. Comparison among Different Systems

**Figure 5:**
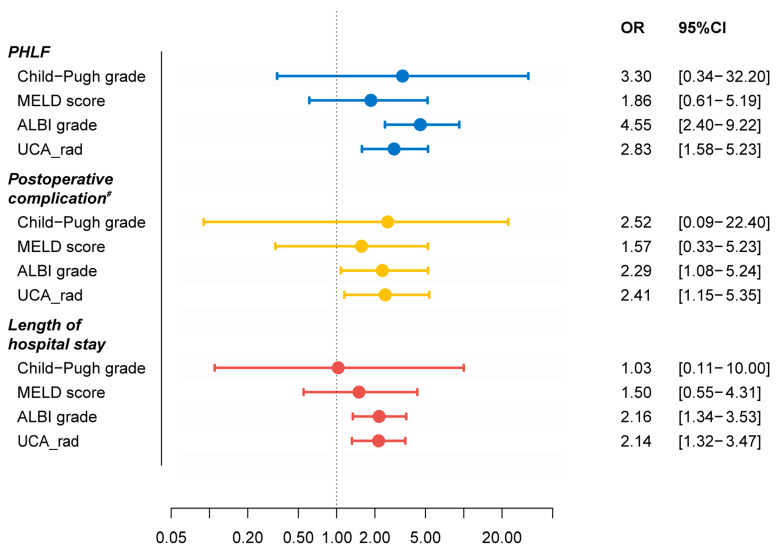
Forest plot showing the odds ratio of the four methods. The reference level was set as Child–Pugh grade A, MELD score ≤ 9, ALBI grade 1, and subgroup 1 in the Child–Pugh score, MELD score, ALBI grade systems, and our unsupervised clustering analysis method. Note: # grade ≥ 2 evaluated by the Clavien–Dindo system. The colors indicate different clinical outcomes. ALBI grade, albumin–bilirubin grade; CI, confidence interval; OR, odds ratio; MELD, model for end-stage liver disease; PHLF, post-hepatectomy liver failure, UCA-rad, unsupervised clustering analysis based on radiomics features.

Compared with subgroup 1, subgroup 2 was associated with a higher risk of PHLF, significant postoperative complications, and longer length of hospital stay, with an OR of 2.83 (95% confidence interval (CI): 1.58–5.23), 2.41(95% CI: 1.15–5.35), and 2.14 (95% CI: 1.32–3.47), respectively (Figure 5).

When comparing the unsupervised clustering method for classification with the preexisting clinical scoring systems, ALBI and the unsupervised clustering method showed to be a significant risk factor for PHLF, postoperative complications, and longer hospital stay (with odds ratios of 4.55 vs. 2.83, 2.29 vs. 2.41, and 2.16 vs. 2.14, respectively), while the Child–Pugh and MELD scoring systems were not significantly different (both *p* > 0.05) (Figure 5).

## 4. Discussion 

This study identified two distinct subgroups among HCC patients scheduled for liver resection using an unsupervised machine learning algorithm based on radiomics features from preoperative gadoxetic-acid-enhanced MRIs. The two subgroups demonstrated significantly different liver function reserves and were associated with different risks of PHLF, postoperative complications, and length of hospital stay. These findings suggest the potential of preoperative gadoxetic-acid-enhanced MRI for liver function reserve evaluation, which may aid the decision making when managing the treatment of HCC patients. To the best of our knowledge, this is the first attempt to use an unsupervised machine learning algorithm on radiomics features of gadoxetic-acid-enhanced MRI to stratify patients into different liver function reserves. 

Patients in subgroup 2 were characterized by older age and marginally more severe cirrhosis, which are two well-established indicators of decreased liver function reserve [43]. The impaired liver function reserve in subgroup 2 was also manifested as a higher rate of ALBI grade 2/3. The ALBI score, which is a novel parameter proposed in recent years, consists of two common liver function biochemical tests: serum albumin and bilirubin [44]. It has proven to be a reliable and accurate alternative to the Child–Pugh system in the objective evaluation of liver function in HCC patients [45]. The newly updated Barcelona Clinic liver cancer (BCLC) staging system has incorporated the ALBI score for objective hepatic reserve estimation and prognosis prediction [46]. Traditional approaches, including laboratory tests (such as ALT, AST, and platelet count) and clinical scoring systems (Child–Pugh and MELD scores), did not show a significant difference between subgroups 1 and 2. 

In the present study, both ICG tests were significantly different between the two subgroups. The strong association between the radiomics features of gadoxetic-acid-enhanced MRI and the ICG test is probably explained by their shared transport pathways [12,47]. The influx and efflux of gadoxetic acid and ICG are both mediated by the hepatocytes via the organic anion transporter (OATP1B3) and the membrane multidrug resistance protein 2 [12,47]. The signal intensity on hepatobiliary phase images of gadoxetic-acid-enhanced MRIs will therefore be closely associated with the ICG test. In fact, signal-intensity-based parameters from the hepatobiliary phase of a gadoxetic-acid-enhanced MRI are the “conventional” method for the quantitative evaluation of liver function [13]. Thus, a decreased function, as indicated by a high ICG test, would result in less MRI signal in the hepatobiliary phase, affecting the radiomics parameters. The findings are in line with those of a pilot study involving 60 patients, showing a similar link between the radiomics features of gadoxetic-acid-enhanced hepatobiliary phase MRI and the ICG test [48]. 

The 107 radiomics features extracted from the gadoxetic-acid-enhanced MRI belonged to morphological features, first-order statistics features, and second-order statistics features [21]. After reproducibility and redundancy evaluation, 37 radiomics features were selected for unsupervised clustering analysis. A majority of these features (27/37) were texture-related features, which evaluated the inter-voxel relationships of the image via grayscale dependence matrices. Eight features (8/37) were based on the first-order histogram, which describes the distribution of the gray levels. In other words, these statistical and textural patterns (so-called hand-crafted radiomics features) [49] do contain biological information for liver function evaluation. The wavelet filter is usually considered a powerful tool to characterize the textural patterns in low- and high-frequency signals [50], and theoretically it can better evaluate the liver function based on gadoxetic-acid-enhanced MR images. Interestingly, in the exploratory stage of this study, unsupervised consensus clustering analysis on the wavelet-transformed radiomics features did not yield a meaningful result. Compared with the standard radiomics technique, deep learning algorithms can detect deep imaging patterns without human inference and its related bias [51]; thus, deep learning may provide a more powerful and robust tool in the evaluation of liver function. Based on hepatobiliary phase images of gadoxetic-acid-enhanced MRIs from 1014 subjects, Park et al. developed a deep learning model to estimate the liver function reserve, showing an area under the receiver operating characteristic curve of 0.93 for predicting ICG-R15 ≥ 20% [52]. 

Subgroup 2 was associated with a higher risk of unfavorable postoperative events such as PHLF, postoperative complications, and longer hospital stays, suggesting a prognostic value of our unsupervised clustering classification. In recent years, several radiomics models have been developed to predict PHLF in HCC patients using preoperative gadoxetic-acid-enhanced MRI showing an area under the receiver operating characteristic curve as high as 0.90 [30,31,32,33]. However, those models were developed from cohorts with a small sample size (<200 patients) without external validation [30,31,32]. In addition, the algorithms used in those studies belonged to supervised machine learning, which is limited by the subjective tags labeled by humans and the delicate model training process [49]. By contrast, unsupervised machine learning excels at detecting hidden patterns within the data and identifying clinically distinct clusters, as shown in this study. 

When evaluating the predictive performance of the three clinical scoring systems and our unsupervised clustering method, ALBI grading and our unsupervised clustering method not only outperformed the Child–Pugh and MELD scoring systems but also showed similar predictive power in the prediction of unfavorable postoperative events. Even so, gadoxetic-acid-enhanced MRI still seems to be a superior approach to the ALBI score, as it can be used to provide the regional liver function information, i.e., the function of the future liver remnant [53]. This can be achieved by delineating the future liver remnant along the planning resection line and extracting the radiomics features for modeling. Those features would be more accurate in the prediction of the postoperative events such as PHLF. This information would be useful for the surgeons when planning an extended hepatobiliary surgery. 

Even though the biological meaning of radiomics features was explained in part by the ICG test in this study, the specific links between radiomics features and the molecular/gene expression levels are lacking. Future studies can be designed to evaluate the link between, for example, OATP1 expression and radiomics features (the work of “radiogenomics”) [54]. Furthermore, although the radiomics features extracted by PyRadiomics have their own formulas, an intuitive understanding of the correlation between these features and liver function classification still lacks. In addition, further investigation is needed to establish the clinical relevance of the identified clusters, given that unsupervised clustering can naturally group image data into different clusters. Although various clinical phenotypes between the two clusters were compared and some significant associations were detected in this study, it is important to assess the meaningfulness of these clusters. 

This study has several limitations. First, the analysis was based on a retrospective cohort of exclusive HCC patients undergoing liver resection at a single medical center from one single MRI scanner using the same sequences. Whether our findings can be extrapolated to a more general patient population or other MRIs remains to be proven by independent cohorts, but we failed in finding an appropriate cohort for external validation. However, a random repeated subsampling cross-validation has been inherently incorporated into the “ConsensusClusterPlus” package used in this study for clustering [42], which might partly ensure the reliability of our results. Second, there might be some confounders for the radiomics features extracted from the non-tumoral liver parenchyma, such as body size, age, and cirrhosis. Our study did not correct these variations as their impact on the radiomics features remains largely unknown. Third, only radiomics features from the hepatobiliary phase were adopted in this study. However, the dynamic changes of radiomics features over the contrast enhancement phases may contain more liver function information. It would be interesting to explore the association between the “delta radiomics features” and liver function/risk of PHLF. Fourth, a comparison between the results of unsupervised machine learning and commonly used supervised machine learning was absent. It would be of interest to compare their predictive efficacy and make new discoveries. Lastly, it is of note to point out that this study is more a preliminary exploring study than a translation study. Our results initially proved the advantages of unsupervised machine learning as a powerful tool for detecting hidden data patterns, grouping patients based on their intrinsic characteristics. Before implementing our results into clinical practice, a great deal of work is required for future research: validation of our findings using external cohorts, automatic segmentation of the non-tumoral part of the liver (or even the future liver remnant), selection of reproducible and informative radiomics features, model interpretation, and integration of the results into daily reports. 

## 5. Conclusions

Unsupervised consensus clustering analysis of the preoperative gadoxetic-acid-enhanced MRIs identified two distinct subgroups of HCC patients who had different liver function reserves and different risks of PHLF. With the increasing use of gadoxetic-acid-enhanced MRI in clinical practice and the advances of artificial intelligence, future research is required to assess and validate the implementation of this unsupervised consensus clustering approach in the management of HCC patients planned for liver resection.

## Figures and Tables

**Table 1:** Clinical characteristics of the whole cohort and the two subgroups identified by unsupervised clustering analysis on radiomics features.

	Overall (*n* = 276)	Subgroup 1 (*n* = 138)	Subgroup 2 (*n* = 138)	*p* Value
Gender				0.382
Female	38 (13.8%)	22 (15.9%)	16 (11.6%)	
Male	238 (86.2%)	116 (84.1%)	122 (88.4%)	
Age (years)				0.003 *
≤55	197 (71.4%)	110 (79.7%)	87 (63.0%)	
>55	79 (28.6%)	28 (20.3%)	51 (37.0%)	
BMI (kg/m^2^)				0.224
≤27	249 (90.2%)	121 (87.7%)	128 (92.8%)	
>27	27 (9.8%)	17 (12.3%)	10 (7.2%)	
Etiology				0.887
HBV	212 (76.8%)	105 (76.1%)	107 (77.5%)	
Non-HBV	64 (23.2%)	33 (23.9%)	31 (22.5%)	
Cirrhosis				0.070
Cirrhosis	146 (52.9%)	65 (47.1%)	81 (58.7%)	
Non-cirrhosis	130 (47.1%)	73 (52.9%)	57 (41.3%)	
Tumor size (cm)				0.278
≤5	130 (47.1%)	70 (50.7%)	60 (43.5%)	
>5	146 (52.9%)	68 (49.3%)	78 (56.5%)	
Resection extent				0.691
Major	80 (29.0%)	38 (27.5%)	42 (30.4%)	
Minor	196 (71.0%)	100 (72.5%)	96 (69.6%)	
Laparoscopy				1.000
No	234 (84.8%)	117 (84.8%)	117 (84.8%)	
Yes	42 (15.2%)	21 (15.2%)	21 (15.2%)	
Blood loss (mL)				0.288
≤400	222 (80.4%)	107 (77.5%)	115 (83.3%)	
>400	54 (19.6%)	31 (22.5%)	23 (16.7%)	
Hepatectomy time (min)				0.753
≤60	227 (82.2%)	112 (81.2%)	115 (83.3%)	
>60	49 (17.8%)	26 (18.8%)	23 (16.7%)	
ALT (IU/L)				0.181
≤42	158 (57.2%)	85 (61.6%)	73 (52.9%)	
>42	118 (42.8%)	53 (38.4%)	65 (47.1%)	
AST (IU/L)				0.181
≤42	158 (57.2%)	85 (61.6%)	73 (52.9%)	
>42	118 (42.8%)	53 (38.4%)	65 (47.1%)	
Platelet (×10^9^/L)				0.164
≤125	96 (34.8%)	42 (30.4%)	54 (39.1%)	
>125	180 (65.2%)	96 (69.6%)	84 (60.9%)	
Child–Pugh grade				0.622
A	272 (98.6%)	137 (99.3%)	135 (97.8%)	
B	4 (1.4%)	1 (0.7%)	3 (2.2%)	
MELD score				1.000
≤9	259 (93.8%)	130 (94.2%)	129 (93.5%)	
>9	17 (6.16%)	8 (5.80%)	9 (6.52%)	
ALBI grade				0.011 *
Grade 1	126 (45.7%)	74 (53.6%)	52 (37.7%)	
Grade 2/3	150 (54.3%)	64 (46.4%)	86 (62.3%)	
ICG-R15 (%)	3.7 (0.3–33.5)	3.2 (0.3–25.3)	4.1(0.3–33.5)	0.002 *
ICG-PDR (%/min)	21.9 (7.3–39.4)	22.9 (8.5–39.4)	21.1 (7.3–35.7)	0.002 *
PHLF				0.001 *
Non-PHLF	211 (76.4%)	118 (85.5%)	93 (67.4%)	
PHLF	65 (23.6%)	20 (14.5%)	45 (32.6%)	
Postoperative complication ^#^				0.030 *
Not significant	241 (87.3%)	127 (92.0%)	114 (82.6%)	
Significant	35 (12.7%)	11 (8.0%)	24 (17.4%)	
Length of hospital stay (days)				0.003 *
≤18	140 (50.7%)	83 (60.1%)	57 (41.3%)	
>18	136 (49.3%)	55 (39.9%)	81% (58.7%)	

Note: # defined by the Clavien–Dindo classification, with Grade ≥ II as significant complications and Grade Ⅰ as not significant complications. Data are expressed as counts with percentages or mean with standard deviation, as appropriate. * *p* < 0.05. ALBI grade, albumin–bilirubin grade; ALT, alanine transaminase; AST, aspartate transaminase; BMI, body mass index; HBV, hepatitis B virus; ICG-PDR, indocyanine green plasma disappearance rate; ICG-R15, indocyanine green retention rate at 15 min; MELD score, model for end-stage liver disease score; PHLF, post-hepatectomy liver failure.

## Data Availability

The original contributions presented in the study are included in the article/Supplementary Material. Further inquiries can be directed to the corresponding authors.

## Supplementary Materials

The following supporting information can be downloaded at: https://www.mdpi.com/article/10.3390/cancers15123197/s1, Table S1: The scanning parameters of the gadoxetic-acid-enhanced MRI used in this study; Table S2: List of the 37 reproducible and non-redundant radiomics features used for unsupervised clustering analysis in this study.
