# Supplementary material for: Unsupervised Machine Learning of MRI Radiomics Features Identifies Two Distinct Subgroups with Different Liver Function Reserve and Risks of Post-Hepatectomy Liver Failure in Patients with Hepatocellular Carcinoma
Source: Cancers (Basel). 2023 Jun 15;15(12):3197. doi: 10.3390/cancers15123197 (PMC10296037; doi:10.3390/cancers15123197)
Supplement: Supplementary file 1 [file cancers-15-03197-s001.zip › cancers-2390751-supplementary.pdf]

Unsupervised machine learning of MRI radiomics features identifies two distinct subgroups with different liver function reserve and risks of post-hepatectomy liver failure in patients with hepatocellular carcinoma

Supplementary materials

Table S1. The scanning parameters of the gadoxetic acid-enhanced MRI

Phases	Flip Angle	Field of View(mm)	Repetition time(ms)	Echo time(ms)	Slice thickness(mm)
Arterial phase	13	400×400	3.42	1.25	2.5
Portal phase	13	400×400	3.42	1.25	2.5
Equilibrium phase	13	400×400	3.42	1.25	2.5
Hepatobiliary phase	30	400×400	3.42	1.25	2.5

Table S2. List of the 37 reproducible and non-redundant radiomics features used for unsupervised clustering analysis in this study.

1	original_shape_Elongation
2	original_shape_Flatness
3	original_firstorder_10Percentile
4	original_firstorder_90Percentile
5	original_firstorder_Energy
6	original_firstorder_InterquartileRange
7	original_firstorder_MeanAbsoluteDeviation
8	original_firstorder_Mean
9	original_firstorder_TotalEnergy
10	original_firstorder_Variance
11	original_glcm_Autocorrelation
12	original_glcm_ClusterProminence
13	original_glcm_ClusterShade
14	original_glcm_DifferenceAverage
15	original_glcm_Id
16	original_glcm_Imc1
17	original_glcm_InverseVariance
18	original_glcm_JointAverage
19	original_glrlm_GrayLevelNonUniformity
20	original_glrlm_GrayLevelVariance
21	original_glrlm_LongRunEmphasis
22	original_glrlm_LongRunHighGrayLevelEmphasis
23	original_glrlm_RunLengthNonUniformityNormalized
24	original_glrlm_RunVariance
25	original_glszm_GrayLevelVariance
26	original_glszm_HighGrayLevelZoneEmphasis
27	original_glszm_LargeAreaEmphasis
28	original_glszm_LargeAreaHighGrayLevelEmphasis
29	original_glszm_SizeZoneNonUniformityNormalized
30	original_glszm_ZonePercentage
31	original_gldm_DependenceEntropy
32	original_gldm_DependenceNonUniformityNormalized
33	original_gldm_DependenceVariance
34	original_gldm_GrayLevelNonUniformity
35	original_gldm_LargeDependenceEmphasis
36	original_gldm_LargeDependenceHighGrayLevelEmphasis
37	original_gldm_SmallDependenceHighGrayLevelEmphasis

27 radiomics features significantly different between subgroup 1 and 2 ($p < 0.05$):

4	original_firstorder_90Percentile
6	original_firstorder_InterquartileRange
7	original_firstorder_MeanAbsoluteDeviation
8	original_firstorder_Mean
9	original_firstorder_TotalEnergy
10	original_firstorder_Variance
11	original_glcm_Autocorrelation
13	original_glcm_ClusterShade
14	original_glcm_DifferenceAverage
15	original_glcm_Id
16	original_glcm_Imc1
17	original_glcm_InverseVariance
18	original_glcm_JointAverage
19	original_glrlm_GrayLevelNonUniformity
20	original_glrlm_GrayLevelVariance
21	original_glrlm_LongRunEmphasis
23	original_glrlm_RunLengthNonUniformityNormalized
24	original_glrlm_RunVariance
27	original_glszm_LargeAreaEmphasis
28	original_glszm_LargeAreaHighGrayLevelEmphasis
29	original_glszm_SizeZoneNonUniformityNormalized
30	original_glszm_ZonePercentage
32	original_gldm_DependenceNonUniformityNormalized
33	original_gldm_DependenceVariance
34	original_gldm_GrayLevelNonUniformity
35	original_gldm_LargeDependenceEmphasis
37	original_gldm_SmallDependenceHighGrayLevelEmphasis

5 radiomics features significantly different between patients with and without PHLF ($p < 0.05$):

2	original_shape_Flatness
13	original_glcm_ClusterShade
29	original_glszm_SizeZoneNonUniformityNormalized
32	original_gldm_DependenceNonUniformityNormalized
36	original_gldm_LargeDependenceHighGrayLevelEmphasis

No radiomics features significantly different between patients with significant and non-significant postoperative complications ($p < 0.05$).

1 Radiomics features significantly different between patients with length of hospital stay ≤ 18 and > 18 days:

	Radiomics features
31	original_gldm_DependenceEntropy
